# Lifespan extension and paraquat resistance in a ubiquinone-deficient *Escherichia coli* mutant depend on transcription factors ArcA and TdcA

**DOI:** 10.18632/aging.100301

**Published:** 2011-03-21

**Authors:** Stavros Gonidakis, Steven E. Finkel, Valter D. Longo

**Affiliations:** ^1^ Integrative and Evolutionary Biology, Department of Biological Sciences, University of Southern California, Los Angeles, CA 90089, USA; ^2^ Molecular and Computational Biology, Department of Biological Sciences, University of Southern California, Los Angeles, CA 90089, USA; ^3^ Andrus Gerontology Center, Department of Biological Sciences, University of Southern California, Los Angeles, CA 90089, USA

**Keywords:** E.coli, aging, lifespan, ArcA, TdcA

## Abstract

We recently reported a genome-wide screen for extended stationary phase survival in *Escherichia coli*. One of the mutants recovered is deleted for *ubiG*, which encodes a methyltransferase required for the biosynthesis of ubiquinone. The *ubiG* mutant exhibits longer lifespan, as well as enhanced resistance to thermal and oxidative stress compared to wt at extracellular pH9. The longevity of the mutant, as well as its resistance to the superoxide-generating agent paraquat, is partially dependent on the hypoxia-inducible transcription factor ArcA. A microarray analysis revealed several genes whose expression is either suppressed or enhanced by ArcA in the *ubiG* mutant. TdcA is a transcription factor involved in the transport and metabolism of amino acids during anaerobic growth. Its enhanced expression in the ubiG mutant is dependent on ArcA. Our data are consistent with the hypothesis that ArcA and TdcA function in the same genetic pathway to increase lifespan and enhance oxidative stress resistance in the *ubiG* mutant. Our results might be relevant for the elucidation of the mechanism of lifespan extension in mutant mice and worms bearing mutations in ubiquinone biosynthetic genes.

## INTRODUCTION

At the population level, *Escherichia coli* maintained in batch culture in the complex, rich medium Luria-Bertani broth (LB) goes through several phases [1]. Briefly, the initial phase of exponential proliferation is followed by a period of stasis during which no further increase in biomass is observed; this period, which is known as stationary phase, is followed by the death phase at the end of which 1-10% of the initial population retains viability as measured either by plate counts or by staining with fluorescent dyes [2]. The progressive loss of viability observed during death phase is reminiscent of the exponential increase in mortality rates over time that characterizes aging in higher organisms [3]. Using the Keio collection in which each non-essential gene has been systematically deleted [4], we found several single-gene mutants that retain 100% viability for longer periods compared to wt [5]. The extended lifespan of the longest-lived strain, which lacks lipoic acid synthase, is dependent on both ArcA and on the bypass of the pyruvate dehydrogenase complex by the pyruvate oxidase – acetyl-CoA synthetase enzyme pair. ArcA is a transcription factor that contributes to the adaptation of *E. coli* to hypoxic conditions by suppressing genes required for oxidative metabolism and activating genes involved in non-oxygen-dependent metabolic functions [6].

One of the long-lived mutants recovered from the screen lacks *ubiG*, which encodes a methyltransferase required for the biosynthesis of ubiquinone [7]. Ubiquinone, also known as coenzyme Q, is a lipid-linked co-factor involved in electron transport in organisms ranging from bacteria to mammals [8]. Its structure consists of a quinone head group that is conserved across species and an isoprenoid hydrophobic tail, the length of which differs among species [7]. A *Caenorhabditis elegans* strain carrying mutations in the gene CLK-1 has been shown to exhibit lengthened embryonic and postembryonic development, as well as extended lifespan [9]. Several years after this initial observation, it was found that while wild-type worms carry ubiquinone in their inner mitochondrial membranes, CLK-1 mutants accumulate demethoxy-ubiquinone [10], raising the possibility that the biosynthesis of ubiquinone and lengthened lifespan are causally related in this mutant. More recently, mice lacking a single copy of mCLK-1, the mouse homolog of CLK-1 were also shown to survive longer than wt [11]. Hence, our findings extend the apparent evolutionary conservation of the regulation of lifespan by ubiquinone to the bacterial domain. Here, we investigate the molecular mechanism of lifespan extension in the *ubiG* mutant, which could be relevant for the study of the long-lived mouse and worm CLK-1 mutants.

## RESULTS

### ArcA-dependent lifespan extension in the *ubiG* mutant

**Figure 1.**
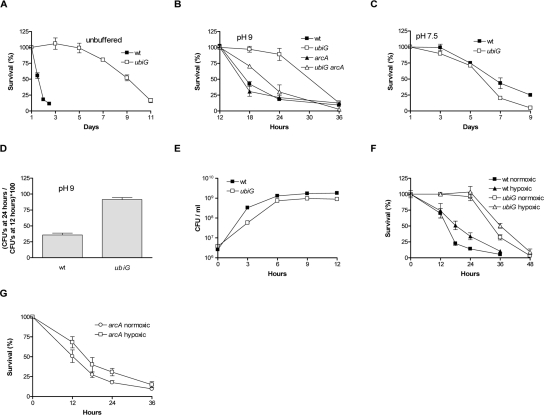
The *ubiG* mutant survives longer than wt (**A-C**) Survival curves of the indicated strains under the shown conditions. (**D**) Fraction of surviving cells at 24 hours in stationary phase for wt and the *ubiG* mutant in the MG1655 genetic background. (**E**) Growth curves of the shown strains. (**F**) Survival of wt and the *ubiG* mutant under normoxia or hypoxia. (**G**) Survival of the *arcA* mutant under normoxia or hypoxia.

The excretion of amines, as a by-product of the metabolism of amino acids in LB, results in an alkaline pH (approximately 9) as a wt strain reaches stationary phase in this medium [12]. On the other hand, the *ubiG* strain reaches a stationary phase pH of approximately 7 (data not shown). Also, the saturating cell density of the wt strain is approximately 1.5 × 10^9^ colony forming units (CFU) per ml, while the *ubiG* mutant reaches a density of approximately 8 × 10^8^ CFU / ml (Figure 1E). The *ubiG* strain survives considerably longer than wt when no changes are made in the media of the two strains (Figure 1A). To dissect the effect of differences in stationary phase pH and cell density on the observed lifespan extension, we equalized stationary phase pH to 9 using the biological buffer AMPSO and stationary phase cell density to 1.5 × 10^9^ CFU / ml for both strains. Though diminished in comparison to the unbuffered experiment, lifespan extension in the *ubiG* strain under these conditions is still significant (Figure 1B). On the other hand, when stationary phase cell density is equalized to 1.5 × 10^9^ CFU / ml and stationary phase pH is equalized to 7.5 using the biological buffer HEPES, the lifespan of the *ubiG* strain is similar to that of wt (Figure 1C). Therefore, the *ubiG* mutation causes a lifespan extension only in an alkaline environment and we performed all subsequent experiments under these conditions. It is important to note that *E. coli* is exposed to alkaline pH in one of its secondary ecological habitats, seawater [13]. The growth curves of wt and the *ubiG* strain demonstrate that both strains enter stationary phase (cease proliferating) at approximately the same time (Figure 1E). Therefore, the observed lifespan extension in the *ubiG* mutant is not a result of delayed entry into stationary phase. Lack of *ubiG* also causes extended survival in the commonly used genetic background MG1655 (Figure 1D). It is worth noting that the stationary phase survival of the *ubiE* and *ubiF* mutants is intermediate between that of wt and the *ubiG* mutant (data not shown).

Prompted by our observations with the *lipA* and *lpdA* strains [5], we tested the effect of *arcA* deletion on the survival of the *ubiG* mutant. Lack of *arcA* has no effect on the survival of wt, but causes a significant shortening of the lifespan of the *ubiG* strain, rendering it closer to that of the wt strain (Figure 1B). Therefore, the hypoxia-inducible transcription factor ArcA is required for the fully extended lifespan of the *ubiG* mutant. Also, we have previously shown that a constitutively active *arcB* allele (the upstream activator of ArcA) results in an ArcA-dependent lifespan extension [5]. Based on these results, we tested the effect of hypoxia on the survival of the two strains, by incubating them in conical flasks with different culture:air ratios (see Methods). Confirming previous findings [14], we found that the survivorship of the wt strain under hypoxic conditions is approximately 2-fold higher compared to normoxia at both 18 and 24 hours (Figure 1F). On the other hand, hypoxia causes an approximately 50% increase in survivorship only at one time-point during the survival experiment of the *ubiG* strain (36 hours, Figure 1F). We also found that the survival of the *arcA* mutant is not different under hypoxic and normoxic conditions, based on four independent replicates per condition, analysed using the student's t test at a signifiance level of p<0.05 (Figure 1G). The dependence of the fully extended lifespan of the *ubiG* mutant on the hypoxia-inducible transcription factor ArcA, as well as the decreased magnitude of the hypoxia-induced lifespan extension in the *ubiG* mutant compared to wt are consistent with the hypothesis that the genetic simulation of hypoxic conditions in the *ubiG* strain is partially responsible for the observed lifespan extension.

### Physiological changes in the *ubiG* mutant

**Figure 2.**
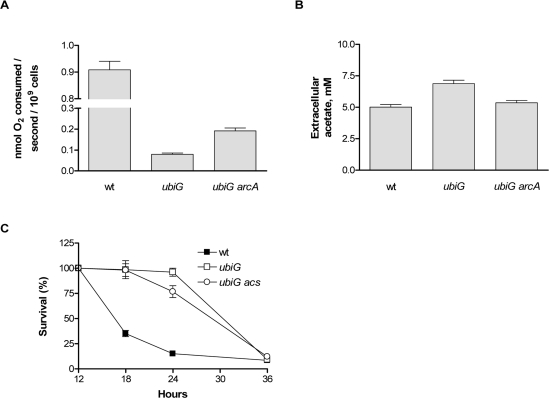
ArcA-regulated physiological changes in the *ubiG* strain (**A**) Rate of oxygen consumption of the shown strains in stationary phase. (**B**) Extracellular acetate accumulation of the shown strains at stationary phase. (**C**) Survival curves of the shown strains.

Ubiquinone mediates the transport of electrons from several electron donors to oxygen and other electron acceptors, such as nitrate in the electron transport chain of *E. coli* [15]. Consistent with these observations, we found that the *ubiG* mutant consumes oxygen at a rate that is one order of magnitude lower than wt during stationary phase. In the absence of *arcA*, the rate of oxygen consumption of the mutant doubles (Figure 2A). Therefore, it appears that the reduced rate of oxygen consumption in the *ubiG* strain is mostly due to the impaired synthesis of ubiquinone and partly due to the action of ArcA, presumably on gene expression.

Acetate is one of the four metabolic by-products of *E. coli* under fermentative conditions, along with lactate, ethanol and succinate [16]. Since the *ubiG* mutant appears to adopt a hypoxia-like metabolism and because we previously showed that acetate production and uptake are required for the extended lifespan of the *lipA* strain [5], we measured the concentration of extracellular acetate in the *ubiG* strain and found it to be higher than that of wt. Deletion of *arcA* reverts the extracellular acetate concentration of the mutant back to wt levels (Figure 2B). Acetyl-coA synthetase, encoded by *acs*, is responsible for the uptake of acetate at extracellular concentrations lower than 10 mM and its conversion to acetyl-CoA [17]. Deletion of *acs* does not affect the lifespan of the *ubiG* mutant (Figure 2C); hence acetate uptake is not required for the longevity of this strain.

**Figure 3.**
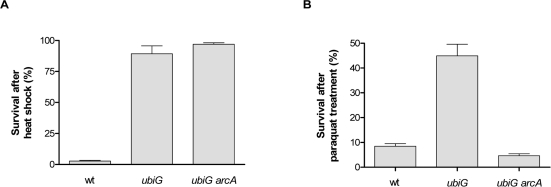
The *ubiG* mutant is stress-resistant (**A**) Survival of the shown strains after a 5′ incubation at 54°C. (**B**) Survival of the shown strains after a 12-hour incubation with 300 μM paraquat.

Long-lived organisms in several model systems have been shown to be resistant to thermal and oxidative stress [18]. The *ubiG* mutant is more resistant than wt both to heat shock and to treatment with the superoxide-generating agent paraquat in stationary phase. The paraquat resistance of the mutant is entirely dependent on ArcA, whereas the heat shock resistance is independent of ArcA (Figure 3). Note that ArcA also contributes to the paraquat resistance of the wt strain, albeit to a much smaller extent compared to the *ubiG* mutant. Therefore, ArcA regulates the lifespan, rate of oxygen consumption, extracellular acetate accumulation and paraquat resistance in the *ubiG* mutant.

### Transcriptional regulation of lifespan

Since ArcA is a transcription factor, we performed a microarray analysis in order to explore its effect on stationary phase gene expression in the *ubiG* strain. The complete dataset is available as an online supplementary file. Hundreds of genes were found to be both significantly downregulated in the *ubiG* strain compared to wt and also significantly upregulated in the *ubiG arcA* strain compared to the *ubiG* strain. In order to communicate the function of these genes more effectively, we used the groups suggested in the Kyoto encyclopedia of genes and genomes [19]. As shown in Table 2, ArcA causes the downregulation of 16 different functional groups of genes in the *ubiG* mutant. Among them, the group of oxidative phosphorylation, an observation consistent with the aforementioned role of ArcA in suppressing oxygen consumption in the *ubiG* mutant (Figure 2A).

Although ArcA predominantly suppresses the expression of genes required for oxidative metabolism, a small number of genes / operons are known to be activated by this transcription factor in response to hypoxia [6]. We found no gene ontology group to be specifically upregulated by ArcA in the *ubiG* mutant.

**Figure 4.**
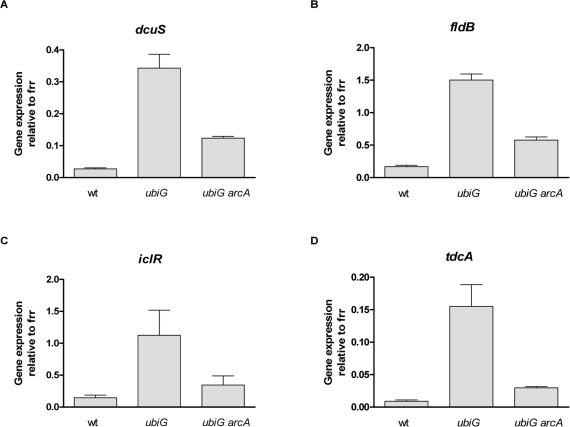
ArcA enhances the expression of several genes in the *ubiG* mutant (**A-D**) Expression of the shown genes relative to *frr* in the respective strains. The expression of the ribosome recycling factor encoded by *frr* was similar across the tested strains.

However, several genes displayed both a higher-than-2-fold upregulation in the *ubiG* strain compared to wt and a higher-than-2-fold downregulation in the *ubiGarcA* strain compared to the *ubiG* strain. We chose to confirm the expression of seven of those genes by RT PCR, selecting these genes based on their involvement in well-described metabolic pathways. As shown in figure 4 and confirming the microarray findings, the expression of *dcuS*, *fldB*, *iclR* and *tdcA* is enhanced in the *ubiG* mutant by ArcA. The expression of *gatC*, *feoB* and *cybB* displayed a similar behavior (data not shown.)

**Figure 5.**
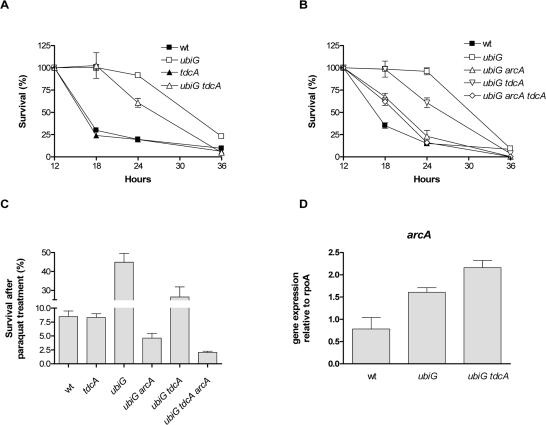
ArcA enhances lifespan and stress resistance through TdcA (**A**, **B**) Survival curves of the shown strains. (**C**) Survival of the shown strains after a 12-hour incubation with 300 μM paraquat. (**D**) Expression of *arcA* relative to *rpoA* in the strains indicated. The expression of the RNA polymerase encoded by *rpoA* was similar across the tested strains.

In order to explore any effects of ArcA-regulated gene expression on the lifespan of the *ubiG* strain, we tested the effect of the deletion of the ArcA-upregulated genes on the survival of the strain. Deletion of *dcuS*, *fldB*, *gatC*, *feoB*, *cybB* or *iclR* has no effect on the survival of the mutant (data not shown). On the other hand, deletion of *tdcA* causes a partial reversion of the extended lifespan of the *ubiG* mutant, while the *tdcA* strain has a stationary phase survival similar to wt (Figure 5A). TdcA is a transcription factor regulating the anaerobic metabolism of serine and threonine [20]. We next explored the genetic interaction between ArcA and TdcA in the regulation of the lifespan and paraquat resistance of the *ubiG* mutant. As shown in figure 5B, the *ubiG arcA tdcA* mutant has a similar survival to the *ubiG arcA* strain, consistent with the hypothesis that ArcA and TdcA function in the same pathway to extend the lifespan of the *ubiG* mutant. TdcA also contributes partially to the paraquat resistance of the *ubiG* strain, since the *ubiG tdcA* mutant is more resistant than wt, but less resistant than the *ubiG* strain to a stationary phase paraquat treatment; TdcA has no effect on the paraquat resistance of wt (Figure 5C). The *ubiG arcA tdcA* mutant displays similar paraquat resistance to the *ubiG arcA* strain. Therefore, the data presented in figures 5B and 5C are pointing towards a single genetic pathway formed by the transcription factors ArcA and TdcA enhancing both stationary phase survival and paraquat resistance in the *ubiG* mutant. Furthermore, the regulation of both paraquat resistance and lifespan by a common genetic pathway suggests that these two phenotypes are physiologically interdependent.

To test whether the expression of *arcA* is under the control of TdcA, we measured the expression of *arcA* in the wt, *ubiG* and *ubiG tdcA* strains. The expression of *arcA* is elevated in the *ubiG* mutant compared to wt and this elevation is not affected by the absence of TdcA (Figure 5D); therefore, the expression of arcA does not seem to be under the control of TdcA. The elevated expression of *arcA* in the *ubiG* mutant might be due to the derepression of the ArcB-ArcA phosphorelay in the absence of fully-formed ubiquinone [21].

## DISCUSSION

**Figure 6.**
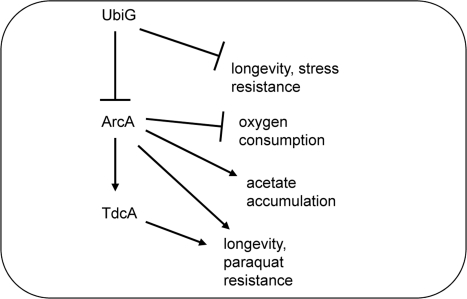
Model for the regulation of lifespan and stress resistance by UbiG in *E. coli.* The hypoxia-induced transcription factor ArcA contributes to the enhanced lifespan and paraquat resistance observed in the absence of *ubiG* in part by enhancing the expression of *tdcA*, a transcription factor involved in the metabolism of serine and threonine during anaerobic growth.

We previously reported a genome-wide screen for extended stationary phase survival in *E. coli* and showed that the hypoxia-inducible transcription factor ArcA and the Acs-PoxB bypass of the pyruvate dehydrogenase complex independently contribute to the longevity of a lipoic acid synthase mutant [5]. The *ubiG* mutant, lacking a gene required for ubiquinone biosynthesis, was also found to be long-lived based on that screen. Here we show that the extended lifespan of this mutant is also dependent on ArcA, but is independent of acetate uptake. ArcA also regulates resistance to the superoxide-generating agent paraquat, oxygen consumption, and extracellular acetate accumulation in the *ubiG* strain. A microarray analysis revealed that 16 gene ontology groups are suppressed by the action of ArcA in the *ubiG* mutant. Furthermore, several genes were found to be upregulated by ArcA in this strain. One of those genes, *tdcA*, contributes to the longevity and paraquat resistance of the *ubiG* strain. Epistasis experiments revealed that ArcA extends the lifespan and enhances the paraquat resistance of this mutant by directly or indirectly activating the transcription of *tdcA* (Figure 6).

Prior to our genetic screen, three studies had reported genetic or environmental interventions that extended the stationary phase survival of *E. coli*. Loss of the toxin-antitoxin *hipBA* locus was shown to increase survival and enhance resistance to hydrogen peroxide [22]. Addition of alcohols to stationary phase cultures was also shown to cause a lifespan extension [23]. Finally, a microscopy-based screen of a transposon-mutagenized *E. coli* population identified a strain lacking the gene *rssB* as longer-lived and more resistant to heat, oxidative and osmotic stress than wt [24]. Since the primary known function of RssB is to control the stability of the master regulator of stationary phase gene expression, RpoS, the authors of the last study hypothesized that ectopic stabilization of the latter is the most likely molecular mechanism behind the observed phenotypes of the *rssB* mutant. Vulic and Kolter [23] also attributed the alcohol-induced lifespan extension they observed to an RpoS-dependent alteration of stationary phase physiology.

**Table 1.** List of strains

Name	Genotype	Source
BW25113	*rrnB3 lacZ4787*Δ *hsdR514* (*araBAD*)Δ *567* (*rhaBAD*)Δ *568 rph*-*1*	Ref. 4
*ubiG*	BW25113 *ubiG*::kan	Ref. 4
*arcA*	BW25113 *arcA*::kan	Ref. 4
*tdcA*	BW25113 *tdcA*::kan	Ref. 4
MG1655	F- lambda- *ilvG*- *rfb*-50 *rph*-1	*E. coli* Genetic Stock Center
SG004	MG1655 *ubiG*::kan	This study
SG013	BW25113 *ubiG*::FRT *arcA*::kan	This study
SG038	BW25113 *ubiG*::FRT *tdcA*::kan	This study
SG039	BW25113 *ubiG*::FRT *arcA*::kan *tdcA*::kan	This study
SG040	BW25113 *ubiG*::FRT *acs*::kan	This study

**Table 2.** Gene ontology groups (according to KEGG) suppressed by ArcA in the *ubiG* mutant. q values are shown

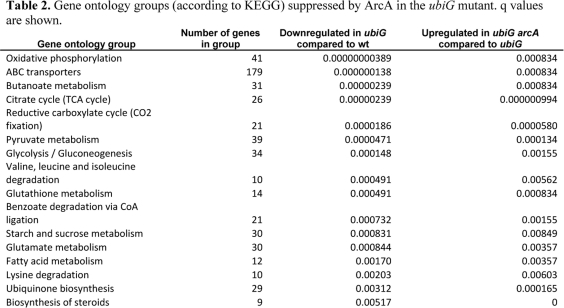

In a previous report, we found that ArcA was required for maximal lifespan extension in a *lipA* mutant, but could not identify the downstream lifespan-enhancing effect of this protein. In this study, we are showing that ArcA is also required for the longevity of the *ubiG* mutant, which lacks one of the methyltransferases required for the biosynthesis of ubiquinone [7]. Incubation under hypoxic conditions was previously shown to extend the stationary phase survival of an *E. coli* population [14]. We confirmed this finding and found that the magnitude of hypoxia-induced lifespan extension is significantly diminished in the *ubiG* strain compared to wt (Figure 1F). Thus, it is possible that hypoxia and the lack of *ubiG* induce a common set of physiological changes that lead to extended lifespan, ArcA being the transcription factor mediating part of those changes. This is further supported by the finding that ArcA is required for hypoxia-induced lifespan extension (Figure 1G). We found that ArcA controls the rate of oxygen consumption, extracellular acetate accumulation, and paraquat resistance in the *ubiG* strain (Figures 2, 3). However, we did not test whether any of those ArcA-controlled physiological changes are causally related to lifespan extension.

In an effort to draw causal relationships between the physiological changes and the lifespan extension induced by ArcA, we turned to the analysis of gene expression, since ArcA is a transcription factor. We used microarrays to measure the expression of all protein-coding genes in wt and the *ubiG* and *ubiG* arcA mutants in stationary phase and found 16 gene ontology groups to be suppressed by ArcA in the *ubiG* strain (Table 2). Consistent with the published literature [6], ArcA suppressed genes belonging to the TCA cycle gene ontology group. Extending our finding of ArcA-mediated suppression of oxygen consumption in the *ubiG* strain (Figure 2A), ArcA was also found to suppress genes belonging to the oxidative phosphorylation group. Interestingly, ArcA also suppresses genes belonging to the ubiquinone biosynthesis functional group, indicating that even in a mutant lacking a ubiquinone biosynthesis gene, the suppression of ubiquinone biosynthesis is partially mediated by ArcA.

A combination of microarray analysis and computational prediction of promoters likely to be regulated by ArcA (based on the sequence of ArcA binding sites derived from footprinting experiments) identified 55 hitherto unknown ArcA-regulated operons [25]. Although the authors of that study used a different functional classification, several cellular functions regulated by ArcA according to that analysis were also identified in our experiments (Table 2), such as amino acid biosynthesis and metabolism, carbon compound catabolism and energy metabolism. Furthermore, our results provide evidence for ArcA regulating processes not previously associated with this transcription factor, such as the expression of ABC transporters. It is important to note that our results describe the effect of ArcA during stationary phase at pH 9 in the *ubiG* strain, whereas Liu and De Wulf studied the effect of ArcA on a wt strain during logarithmic phase under hypoxia in LB supplemented with xylose and a constant pH of 7.4 [25].

Although ArcA predominantly exerts a negative effect on its targets, it also activates the transcription of several operons [25]. According to our microarray results, the transcription of several genes is activated by ArcA in the *ubiG* strain; we confirmed these expression changes in a subset of those genes (Figure 4). One of those genes, *tdcA* is required for maximal lifespan extension and paraquat resistance in the *ubiG* mutant (Figure 5). TdcA is the transcriptional activator of the *tdc* operon, which is involved in the transport and utilization of serine and threonine during anaerobic growth [26]. Both ArcA and Fnr, the second major regulator of anaerobic gene expression, are known to independently activate the transcription of *tdcA* in a wt strain under anaerobic conditions [27]. The authors of the latter study attributed the effects they observed on the action of an unidentified metabolite, the production of which is indirectly facilitated by ArcA or Fnr.

The activation of the transcription of *tdcA* by ArcA, as well as the similar lifespan and paraquat resistance phenotypes of the *ubiG arcA* and the *ubiG arcA tdcA* mutants points towards a model where ArcA contributes to the extended lifespan and enhanced paraquat resistance of the *ubiG* strain (Figure 6). Based on the known function of the *tdc* operon, it is possible that TdcA enhances lifespan and paraquat resistance by activating the uptake and utilization of serine and/or threonine. Among seven amino acids studied, serine is the first one to be consumed from a wt strain during incubation in a complex medium similar to LB [28]. Threonine also disappears from the medium by the onset of stationary phase. It is possible that the metabolic impairment caused by the mutation in *ubiG* retards the uptake and utilization of these two amino acids in the *ubiG* mutant, making them available in the extracellular medium for longer periods compared to wt. According to this hypothesis, ArcA activates the transcription of TdcA, which in turn activates the transcription of TdcA and TdcC, allowing the uptake and utilization of serine and threonine, thus leading to extended stationary phase survival and enhanced paraquat resistance. This hypothesis could be tested by analyzing the effect of ArcA and TdcA on the amino acid consumption profile of wt and the *ubiG* mutant.

Hypoxia-inducible factor 1 (HIF-1) is a transcription factor regulating the response to shortage of oxygen in organisms ranging from nematode worms to humans [29, 30]. Although they do not share a sequence homology, ArcA can be thought of as the functional homolog of HIF-1 in *E. coli*. HIF-1 was recently implicated in the regulation of lifespan in *Caenorhabditis elegans*. Worms lacking VHL-1, a protein required for the degradation of HIF-1 under normoxic conditions live longer than wt worms [31]. Extending this finding, Zhang et al., found that overexpression of HIF-1 leads to lifespan extension in the same model organism [32].

The mechanism of lifespan extension in worms and mice carrying mutations in CLK-1, a protein implicated in ubiquinone biosynthesis in both organisms, remains unclear. Experiments exploring the metabolic rate of worms carrying mutations in CLK-1 have not produced a clear answer, with findings ranging from no change to a small decrease [33]. Several studies have reported decreased oxidative damage arising from reactive oxygen species in clk-1 mutant worms (e.g. [34]). However, a number of genetic and environmental interventions affecting ROS production and oxidative damage in *C. elegans* have produced inconsistent results [35]. It is still possible that the clk-1 mutation affects lifespan through the effect of reduced ROS production on multiple signaling pathways [33]. Although mice are not normally exposed to hypoxic conditions in the wild, a recent study implicated epidermal sensing of oxygen by VHL and HIF-1 in the physiological response of mice in response to hypoxia [36]. Our results raise the possibility that the molecular mechanism of lifespan extension in worms and mice with impaired ubiquinone biosynthesis involves the genetic response to hypoxia, which might represent a metabolic adaptation required for the extended lifespan of evolutionarily distant model organisms.

## METHODS

### Strains and genetic manipulations

The wild-type *Escherichia coli* strain BW25113, a K-12 derivative, and its respective single-gene knock-outs were provided by the Keio collection [4]. Each gene is knocked out by the insertion of a kanamycin-resistance cassette. To create strains deleted for multiple genes, the kanamycin cassette was excised using FLP-mediated recombination, resulting in deletions carrying only a single FRT site (denoted, for example *ubiG*::FRT in Table 1), as previously described [37], with the only difference that the non-selective incubation took place at 37°C and not at 43°C. Kanamycin alleles were transduced by bacteriophage P1 using standard techniques and the correct insertion was verified by PCR using primer K1 described in [4], along with a locus-specific primer annealing to a sequence upstream of the disrupted locus. Genomic sequence information was obtained from the “Profiling of the *E. coli* Chromosome” web site (www.shigen.nig.ac.jp/ecoli/pec/index.jsp). All strains used in the study are shown in Table 1.

### Survival experiments

LB medium consisted of 1% bacto tryptone, 0.5% yeast extract and 0.5% NaCl w/v. Cultures were inoculated 1:1000 (vol:vol) using an overnight culture created by inoculating 2-3 colonies from an LB plate to 1 ml of LB. Cultures were grown in 3 ml's of LB in 16-mm diameter test tubes rotating orbitally at 220 rpm for 12 hours, at which point cell density was adjusted to ~1.5 × 10^9^ CFU per ml by resuspending a pellet containing the desired number of cells in cell-free spent medium of the same strain for the long-lived strains with reduced saturation cell density. AMPSO was added to 100 mM to achieve a stationary phase pH of 9. Due to the differences in stationary phase pH reached spontaneously by each strain, the pH of the cultures had to be adjusted accordingly for each strain. Spontaneous and adjusted pH was quantified using a pH electrode and pH test strips. All cultures were grown and maintained at 37°C and 70% relative humidity and colony-forming units (CFU) were enumerated over time by removing an aliquot, serially diluting in 0.5% NaCl, followed by colony enumeration after plating on LB plates that were incubated at 37°C. For the hypoxia experiments, 30-ml cultures were incubated for 12 hours in orbitally shaking 125-ml Erlenmeyer flasks. The resulting stationary phase culture was split to two different cultures in 25-ml flasks, each with 100 mM AMPSO; one 5-ml culture (‘normoxic’) and one 28-ml culture (‘hypoxic’); note that the 25-ml notation is nominal, since the flask can actually hold approximately 30 ml. CFU's were enumerated over time as described above.

### Stress resistance

For the heat shock experiment, cultures were processed as described above with AMPSO, returned to the incubator and 4 hours later (16 hours after inoculation) subjected to either a 5-minute incubation in a 54°C waterbath without shaking and CFU enumerated before and after the treatment.

For the paraquat experiment, cultures were processed as described above with AMPSO and paraquat (methyl viologen dichloride hydrate, Sigma-Aldrich) was added to 300 μM at the time of processing (12 hours after inoculation). CFU were enumerated before and after a 12-hour incubation at 37°C.

### Oxygen consumption and acetate quantification

Oxygen consumption measurements were performed with 2-ml of culture stirred by a magnetic stir bar in a 37°C waterbath using a Clark-type electrode. Conversion to nanomoles of oxygen consumed was done by assuming that the liquid culture contains the same amount of oxygen as water equilibrated with 21% oxygen in 1 atmosphere pressure, which is 5.02 μl/ml (manufacturer's manual) and was further normalized by the number of CFU's present. Data were recorded until a straight line trace was obtained indicating that a steady state of oxygen consumption had been reached. Extracellular acetate concentration was quantified on cell-free samples obtained by centrifugation using the R-Biopharm acetic acid kit (catalog number 10148261035) according to the manufacturer's instructions.

### Microarrays and RT PCR

Stationary phase cultures of wt and the *ubiG* and *ubiG* arcA mutants were prepared as mentioned in the ‘survival experiments’ section. Cultures were removed from the incubator 45 min. after the addition of AMPSO and 1-ml aliquots were added to a 95μl ethanol + 5μl water-equilibrated acidic phenol mixture and rapidly centrifuged for 45 seconds at 4°C. RNA was subsequently extracted using the MasterPure complete DNA and RNA purification kit (Epicentre Biotechnologies, catalog number MC85200) according to the manufacturer's instructions. The MicrobExpress kit (Ambion) was used to enrich the resulting RNA for mRNA, using the manufacturer's instructions. 200 ng of MicrobExpress RNA was used to generate double stranded cDNA using Ambion's MessageAmp II-Bacteria kit. This cDNA was subsequently used as a template for an *in vitro* transcription reaction to synthesize biotin-labeled amplified RNA in the presence of 10 mM biotin-11-CTP and 10 mM biotin-16-UTP. The *in vitro* transcription reaction volume was 20 μl and incubated for 14 hours. Fragmentation of the labeled, amplified RNA was performed according to the instructions found in the Affymetrix GeneChip Eukaryotic Expression Manual. 4 μg of the fragmented target was hybridized at 45°C with rotation for 16 hours at 60 rpm to probe sets present on an Affymetrix *E. coli* 2.0 array. The microarrays were washed, stained (streptavidin, phycoerythrin) and scanned on an Affymetrix fluidics station 450. Arrays were processed at the UC Irvine DNA & protein microarray facility.

2 microarrays were analysed per strain. After imaging process, the expression of each sample is represented by a CEL file, which includes the fluorescence intensities of all probes. We processed the microarray data using the invariant-set normalization [38] followed by median polish summarization [39], and obtained expression for each probe set. The computation was carried out in statistical software R using the Affy package [40]. The interpretations of gene expression changes were based on the gene set enrichment analysis. It determines whether a pre-defined set of genes shows significantly up- or down-regulation compared to other gene sets. We downloaded the KEGG pathway database from ftp://ftp.genome.jp/pub/kegg/pathway/organisms/eco/. It provides manually annotated metabolic and signaling pathways. We selected 102 well-established *Escherichia coli* pathways to define KEGG gene sets. We carried out the enrichment analysis followed by the Wilcoxon scoring scheme as previously described [41]. Briefly, based on the expression differentiation between two samples, we compared the fold changes of KEGG gene set Si against the background expression changes denoted by G-Si, where G is the union of all KEGG gene sets. We adopted the Wilcoxon rank test to calculate p-values (one-sided) and assigned the final significance scores after multiple testing correction using the “qvalue” package [42] in R.

To test the expression of *dcuS*, *fldB*, *iclR*, *tdcA* and *arcA*, RNA was extracted as described above. 3 μg of RNA were used per reverse transcription reaction using Superscript III reverse transcriptase (Invitrogen) and random hexamers as primers according to the manufacturer's instructions. 50 ng of reverse-transcribed RNA were then used as substrate for real-time PCR. The expression level of three different housekeeping genes was measured (*rpoA*, *frr* and *dnaA*) and the one showing similar expression levels among all tested strains was used for normalization. Standard curves were constructed for each assayed transcript and used for quantification. The primers used are shown in Table S2.

## SUPPLEMENTARY TABLES


