# Supplementary material for: Lifespan extension and paraquat resistance in a ubiquinone-deficient Escherichia coli mutant depend on transcription factors ArcA and TdcA
Source: Aging (Albany NY). 2011 Mar 21;3(3):291–303. doi: 10.18632/aging.100301 (PMC3091522; doi:10.18632/aging.100301)
Supplement: Supplementary file 1 [file aging-03-291-s001.pdf]

40. Gautier L et al. Affy--analysis of Affymetrix GeneChip data at the probe level. Bioinformatics. 2004; 20:307-315.
41. Cheng C et al. Significant and systematic expression differentiation in long-lived yeast strains. PLoS One. 2007. 2:e1095.
42. Storey JD and Tibshirani R.. Statistical significance for genomewide studies. Proc Natl Acad Sci U S A. 2003; 100:9440-9445.

SUPPLEMENTARY TABLES

Data of **Table S1. Complete results of microarray experiment** are found in full text version of this manuscript.

Table S2. List of primers used for RT PCR

Name	Sequence
frr 5’	TCGTGGTGAAGCAGAACAAG
frr 3’	ATCGTCGTCTTCGCTGATCT
rpoA 5’	CAACCATTCTGGCTGAACAA
rpoA 3’	GCGGACAGTCAATTCCAGAT
dcuS 5’	ACTGTCGACGCTAAGCCACT
dcuS 3’	TGAAATGGCACCGATGATAA
fldB 5’	ACGGTTCCAGCACCTGTTAC
fldB 3’	CCCAGTCTTCCTGGATTTCA
iclR 5’	AATGGAAGAGTCTGGCGAAA
iclR 3’	CGCTCAGTTGGGCTAAAAAG
tdcA 5’	CGGTGGTGGAAGTCTCATTT
tdcA 3’	ACCAATCGCAAAATCCAGTC
arcA 5’	CAGACCCCGCACATTCTTAT
